# Disrupted Brain Structural Connectivity Network in Subcortical Ischemic Vascular Cognitive Impairment With No Dementia

**DOI:** 10.3389/fnagi.2020.00006

**Published:** 2020-01-29

**Authors:** Linqiong Sang, Chen Liu, Li Wang, Jingna Zhang, Ye Zhang, Pengyue Li, Liang Qiao, Chuanming Li, Mingguo Qiu

**Affiliations:** ^1^Department of Medical Imaging, School of Biomedical Engineering, Third Military Medical University, Chongqing, China; ^2^Department of Radiology, Southwest Hospital, Third Military Medical University, Chongqing, China; ^3^Department of Radiology, The Second Affiliated Hospital of Chongqing Medical University, Chongqing, China

**Keywords:** subcortical ischemic vascular cognitive impairment with no dementia, graph theoretical analysis, brain structural network, network-based statistic, topological organization

## Abstract

The alteration of the functional topological organization in subcortical ischemic vascular cognitive impairment with no dementia (SIVCIND) patients has been illuminated by previous neuroimaging studies. However, in regard to the changes in the structural connectivity of brain networks, little has been reported. In this study, a total of 27 subjects, consisting of 13 SIVCIND patients, and 14 normal controls, were recruited. Each of the structural connectivity networks was constructed by diffusion tensor tractography. Subsequently, graph theory, and network-based statistics (NBS) were employed to analyze the whole-brain mean factional anisotropy matrix. After removing the factor of age, gender, and duration of formal education, the clustering coefficients (C_*p*_) and global efficiency (E_*glob*_) were significantly decreased and the mean path length (L_*p*_) was significantly increased in SIVCIND patients compared with normal controls. Using the combination of four network topological parameters as the classification feature, a classification accuracy of 78% was obtained by leave-one-out cross-validation for all subjects with a sensitivity of 69% and a specificity of 86%. Moreover, we also found decreased structural connections in the SIVCIND patients, which mainly concerned fronto-occipital, fronto-subcortical, and tempo-occipital connections (NBS corrected, *p* < 0.01). Additionally, significantly altered nodal centralities were found in several brain regions of the SIVCIND patients, mainly located in the prefrontal, subcortical, and temporal cortices. These results suggest that cognitive impairment in SIVCIND patients is associated with disrupted topological organization and provide structural evidence for developing reliable biomarkers related to cognitive decline in SIVCIND.

## Introduction

Cognitive impairment is frequently induced by the subcortical ischemic vascular disease (SIVD), and it is marked by lacunar infarcts and deep white matter changes (Erkinjuntti, 2003; O’Brien and Thomas, 2015). Subcortical ischemic vascular cognitive impairment with no dementia (SIVCIND) has been considered a “disconnection syndrome” due to the extensive damage to the white matter tracts or U-fibers, which connect cortical and subcortical regions. Early identification of SIVCIND is of great importance since managing these risk factors in a timely manner may prevent disease development and reduce disease progression. However, the definite etiology and pathogenesis of cognitive decline induced by SIVD remains poorly understood.

Some studies have shown that changes in white matter of brain were existed in patients with cognitive impairment induced by SIVD. For instance, decreased fractional anisotropy (FA) was found in several projection fibers and association fibers in SIVCIND patients, including the posterior thalamic radiations, cingulum, and fronto-occipital fasciculus (Lin et al., 2015). Zhou et al. (2011) found decreased FA in several brain regions located in the bilateral frontal, occipital, and temporal cortices in SIVCIND patients compared with normal controls. Another research (Xu et al., 2010) recruited patients with SIVD, who were subdivided into three groups: normal cognition, cognitive impairment with no dementia, and dementia. Significant differences were found in the FA values in whole-brain white matter between normal cognition group and cognitive impairment group; the FA values were also significantly correlated with attention, executive, and memory performance. Although it is incremental in the knowledge of the structural abnormalities in specific regions of the brain, the definite biomarkers which may identify the cognitive decline induced by SIVD remain unclear.

Recently, the increasing studies have shown that many of the important topological organizations of brain networks could be revealed by graph theoretical approaches (Bullmore and Sporns, 2009; He and Evans, 2010; Lopes et al., 2017). Moreover, the topological organization of whole-brain functional and structural networks could be employed to characterize some neurological and psychiatric disorders (Zhang et al., 2011; Shu et al., 2012; Zhu et al., 2016). Notably, one study recruited 127 small vessel disease patients which were comprised of 76 mild cognitive impairment patients and 51 normal cognitive patients; the results showed that global network efficiency was significantly correlated with cognitive state (*p* < 0.01) (Du et al., 2019). Another study (Heinen et al., 2018) considered the relationship between total small vessel disease burden score, global network efficiency and cognition; the results showed that global network efficiency was relevant to the performance on the information processing speed, attention and executive functioning. However, it remains unclear regarding the changes in the structural connectivity network in SIVCIND patients and the power of network parameters identifying SIVCIND patients from controls.

In this study, we aimed to observe alterations in the whole-brain structural connectivity networks in SIVCIND patients and explore the relationship between changes in brain structural organization and cognitive deficits. Here, we hypothesized that the organization of the structural connectivity network would be disrupted and that the changes would be related to cognitive decline. More specifically, we expected that the cognitive impairment caused by SIVD would be associated with altered global parameters of the whiter matter network. In other words, the topological properties of the white matter network may be related to cognitive state. Finally, the study was carried out to estimate whether the feature of topological organization of the brain white matter network could be biomarkers of the cognitive decline induced by SIVD.

## Materials and Methods

### Participants

Twenty-seven Right-handed participants were recruited in the study, including 13 SIVCIND patients and 14 normal controls. Written informed consent was obtained from all subjects. This study was approved by the local Medical Ethics Committee at Third Military Medical University (Chongqing, China) on Human Studies.

According to the criteria suggested by Galluzz, only the SIVCIND patients who had a subcortical WM hyperintensity on T2-weighted imaging and had at least two lacunar infarcts were enrolled in this study (Galluzzi et al., 2005). Moreover, the special inclusion criteria for SIVCIND was insufficient cognitive impairment to meet the DSM-V criteria for dementia, and the Hachinski Ischemic Score (HIS) ≥7 (Sachdev et al., 2014; Yu et al., 2017). For detailed description of inclusion criteria and exclusion criteria for SIVCIND, please see Sang et al. (2018). All patients received baseline evaluations, including complete sociodemographic, and clinical data collection. The performance on cognitive functioning was assessed by several neuropsychological tests including the Mini Mental State Examination (MMSE), the Clinical Dementia Rating (CDR), the Global Deterioration Scale (GDS), the Montreal Cognitive Assessment (MoCA) (Folstein et al., 1975; Hachinski et al., 1975; Reisberg et al., 1982; Warren et al., 1989; Moris, 1993; Cummings et al., 1994; Nasreddine et al., 2005).

Each of the normal controls had well-documented normal cognitive performance and none have any nervous system diseases. The conventional MRI and neuropsychological tests was implemented for each normal control. None had vascular risk factors that could induce cognitive impairment or current or a history of psychiatric illness. Moreover, none of them had brain trauma, brain tumors, systemic disease or other MRI contraindications, such as claustrophobia.

### Data Acquisition

All subjects underwent MRI scanning using a Siemens 3.0 T Trio MRI scanner (Siemens, Erlangen, Germany) at Southwest hospital, chongqing, china. The T1-weighted anatomical images (repetition time = 1900 ms; echo time = 2.52 ms; flip angle = 9°; voxel size = 1 × 1 × 1 mm^3^; slice thickness = 1 mm with no gap; 176 slices; field of view = 260 mm; matrix = 256 × 256) were acquired. Then, the diffusion tensor images [repetition time = 5500 ms; echo time = 93 ms; flip angle = 90°; voxel size = 1.8 × 1.8 × 3 mm^3^; slice thickness = 3 mm with no gap, 40 slices; field of view = 230 mm; matrix = 128 × 128; 64 non-collinear diffusion-weighted gradient direction (b = 1000 s/mm^2^) and one additional unweighted image b0 (*b* = 0 s/mm^2^)] were acquired.

### Network Construction

#### Data Preprocessing

First, we used FSL software^1^ to correct head motion and eddy currents by registering each diffusion-weighted image to the first unweighted b0 image, and the FSL-BET program was applied to remove skull and other non-brain tissue. Subsequently, a deterministic streamline fiber tractography algorithm was performed to obtain each subject’s whole-brain tractography in the native diffusion space (Fiber Assignment by Continuous Tracking algorithm, FACT) implemented in the Diffusion Toolkit software^2^. If the FA value was less than 0.2 and the curvature of path tracing was greater than 45°, the tracking would be terminated.

#### Node Definition

Nodes and edges are two components of a network. The node definition in the structural connectivity network was made according to the AAL template (45 for each hemisphere). In this study, the AAL template was mapped from the standard MNI space to the individual’s native DTI space by inverse transformations from spatial normalization and registration. Detailed information on each brain region in the AAL template is presented in Supplementary Table S1.

#### Edge Definition

An edge in the structural connectivity network was defined when the number of fibers between two regions was equal to or greater than three. If at least three fibers were present, the two regions were considered linked through an edge, and the value of the edge was set to 1. The final value of the edge was obtained by multiplying the mean FA values along the fiber bundles connecting a pair of regions; in this way, the edges revealed the white matter structure. Therefore, the weighted structural connectivity network was constructed for each subject.

**FIGURE 1:**
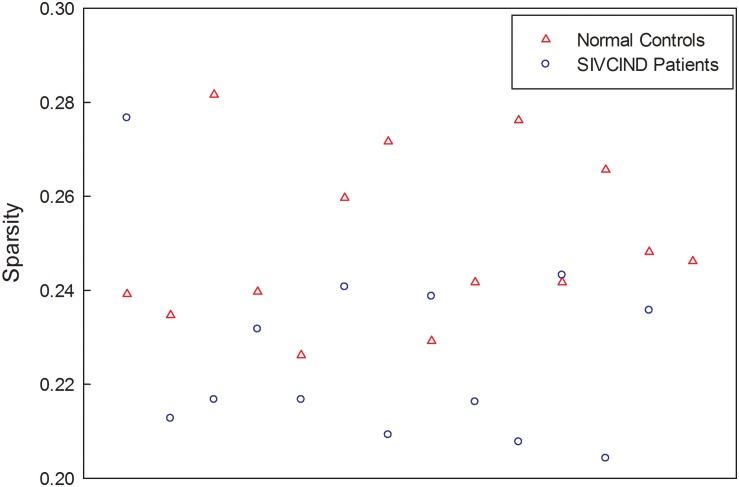
The sparsity of each subject’s brain structural connectivity network.

Finally, the factors of age, gender and formal education duration were removed from each weighted connectivity matrix by regression. The resultant structural network of each subject is a sparse matrix, and the sparsity of each network, ranging from 20 to 28%, is shown in Figure 1.

### Network Analysis

To ensure that all resultant networks had the same number of nodes and edges, the weighted network was thresholded at different levels of sparsity ranging from 5 to 20% in increments of 1%. At each threshold, the network metrics were calculated. Specifically, the global metrics of each structural network were constant and ranged from 14 to 20% (Supplementary Figure S1); hence, the threshold at different levels of sparsity, ranging from 5 to 14%, was employed in this study.

Moreover, we calculated several global network parameters of the weighted brain structural network: the clustering coefficient (C_p_), the mean path length (L_p_), the global efficiency (E_glob_), and the local efficiency (E_loc_). Definitions of these metrics are as follows.

The C_p_ of a network G describes the connectedness of direct neighbors around individual nodes (Watts and Strogatz, 1998). It is expressed as follows (Onnela et al., 2005):

$$C_{p}\,\left(G\right)=\underset{i\in n\,o\,d\,e}{m\,e\,a\,n}\frac{2}{k_{i}\,\left(k_{i}-1\right)}\,\sum_{j,k}{\left(\bar{w_{i\,j}}\,\bar{w_{j\,k}}\,\bar{w_{k\,i}}\right)}^{1/3}$$

where w_ij_ is the weight value of the edges between node i and node j in the network, and k_*i*_ is the strength of node i.

The L_p_ of G quantifies the ability for information transfer in parallel. It is expressed as follows:

$$L_{p}\,\left(G\right)=\frac{1}{N\,\left(N-1\right)}\,\sum_{i\neq j\in G}L_{i\,j}$$

where N is the number of nodes, and L_ij_ is the shortest path length between nodes i and j in the network.

The value E_g_ of G quantifies the E_glob_ of the parallel information transfer in the network. It is expressed as follows:

$$E_{g}\,\left(G\right)=\frac{1}{N\,\left(N-1\right)}\,\sum_{i\neq j\in G}\frac{1}{L_{i\,j}}$$

where N is the number of nodes, and L_ij_ is the shortest path length between nodes i and j in the network.

The E_loc_ of G quantifies the fault tolerance of a network (Achard and Bullmore, 2007). It is expressed as follows:

$$E_{l\,o\,c}\,\left(G\right)=\frac{1}{N\,\left(N-1\right)}\,\sum_{i\neq j\in G}E_{g}\,\left(G_{i}\right)$$

where N is the number of nodes, and G_i_ represents the subgraph which is comprised of the direct neighbors around node i.

Moreover, we calculated the nodal global efficiency E_nodal_glob_ and nodal local efficiency E_nodal_loc_ to quantify the ability of information transfer between a node and all other nodes and its direct neighbors in the network. They are expressed as follows:

$$E_{n\,o\,d\,a\,l\,\_\,g\,l\,o\,b}\,\left(i\right)=\frac{1}{N-1}\,\sum_{i\neq j\in G}\frac{1}{L_{i\,j}}$$

$$E_{n\,o\,d\,a\,l\,\_\,l\,o\,c}\,\left(i\right)=E_{g}\,\left(G_{i}\right)$$

where N is the number of nodes, L_ij_ is the shortest path length between nodes i and j, and G_i_ represents the subgraph which is comprised of the direct neighbors around node i.

The topological parameters of the brain networks were computed using GRETNA toolbox^3^. Furthermore, the area under the curve (AUC) for each network metric (C_p_, L_p_, E_g_, E_bloc_, E_nodal_glob_, and E_nodal_loc_) was calculated to characterize the topological organization of the brain structural network according to previous studies (He et al., 2009).

An SVM was applied to build classification models with a radial basis function as the kernel function, which determines the power of a combination of global topological parameters to predict the subject groups. In this classification model, the predictors were the combination of the four global network metrics (C_p_, L_p_, E_g_, and E_bloc_), and the response was the subject’s group. Furthermore, we performed leave-one-out cross-validation to evaluate the classification accuracy, which guarantees a relatively unbiased estimate of the generalization power of the classifiers to new subjects. Subsequently, the sensitivity and specificity were calculated.

Moreover, the network-based statistics (NBS) approach, a validated non-parametric statistical approach for controlling familywise error in connectome analyses (Zalesky et al., 2010), was applied to identify the altered structural connections in patients. First, the strength of each edge weight in the structural connectivity matrix was compared between the patient group and the control group by using a one-tailed *t*-test. Second, the network components involving the surviving connected edges with an uncorrected *p*-value of 0.005 were retained. Third, the size of the largest network component was computed. Then, the groups were randomly shuffled (10,000 permutations), and the largest network component size was calculated by repeating steps 1, 2, and 3. In this way, an empirical null distribution was generated to evaluate the statistical significance of the network component sizes. Moreover, the corrected *p*-value for a network component with size M, which was found in the correct grouping of controls and patients, was determined by calculating the proportion of the maximal network component that was larger than M in the 10,000 permutations.

In this study, we used circos software^4^ to show significant connections between patients and controls by circular graphical representations (Irimia et al., 2012). In a circular graph, links between pairwise regions are colored by connection type, such as blue representing left intrahemispheric connections, green representing right intrahemispheric connections, and red representing interhemispheric connections. ROIs were grouped into frontal, temporal, parietal, medial temporal, occipital, and subcortical according to Salvador et al. (2005). According to a previous study, the ROIs with a high degree of significant connections (*k* > 1 SD above the mean) were deemed “network hubs” (Lopes et al., 2017).

### Statistical Analysis

The non-parametric permutation test was performed on the AUC of each network metric to identify between-group differences based on a null permutation distribution, which was generated by randomly assigning two groups with the same sizes as the original groups of patients and normal controls. This randomization procedure was repeated for 10,000 permutations, and the between-group differences of each graph metric were calculated to generate a null permutation distribution. The *p*-value was obtained by computing the proportion of differences exceeding the value in the correct grouping of patients and normal controls in the null distribution. A *p*-value lower than 0.05 was deemed to manifest statistical significance. Specially, the effects of age, gender and formal education duration were removed using multiple linear regression before the permutation test.

Furthermore, to identify the relationship between the topological properties of the structural connectivity network and cognitive performance, we performed Person’s correlation between the global topological metrics (C_p_, L_p_, E_g_, and E_bloc_) and the cognitive scores (MMSE and MoCA) in SIVCIND patients. Additionally, we computed the *Z*-scores of each cognitive scores, and the statistical significance level of *p*-value is lower than 0.05.

## Results

### Clinical Statistics

Subject demographics are listed in Table 1. A two-sample *t*-test revealed that the SIVCIND patients showed lower MMSE (*T* = 5.729, *p* < 0.001) and MoCA scores (*T* = 10.294, *p* < 0.001) compared to the normal controls. No significant difference was found between the two groups in terms of gender (χ^2^ = 0.333, *p* = 0.564), age (*T* = −1.092, *p* = 0.285) and duration of formal education (*T* = 1.188, *p* = 0.246).

**TABLE 1:** Demographics and clinical characteristics of the subjects.

	**NC (*n* = 14)**	**SIVCIND (*n* = 13)**	***p*-Value**
Gender (male/female)	8/6	7/6	0.564^a^
Age (years)	58–76 (65.1 ± 5.0)	47–83 (68.3 ± 9.8)	0.285^b^
Education (years)	0–16 (10.4 ± 4.1)	1–16 (8.5 ± 4.1)	0.246^b^
MMSE	22–30 (28.4 ± 2.2)	18–26 (23.5 ± 2.3)	< 0.001^b^
MoCA	21–30 (27.1 ± 2.3)	8–22 (14.7 ± 3.8)	< 0.001^b^
HIS	–	7–14 (8.7 ± 1.4)	
GDS	–	3–5 (3.7 ± 0.5)	
CDR	–	0.5–1 (0.6 ± 0.1)	

*Data are expressed as the min–max (mean ± SD). MMSE, Mini-Mental State Examination; MoCA, Montreal Cognitive Assessment; HIS, Hachinski Ischemic Score; GDS, Global Deterioration Scale; CDR, Clinical Dementia Rating. ^*a*^*p*-value obtained using Pearson’s Chi-squared test. ^*b*^*p*-value obtained using a two-sample *t*-test.*

### Altered Global Topological Properties

**FIGURE 2:**
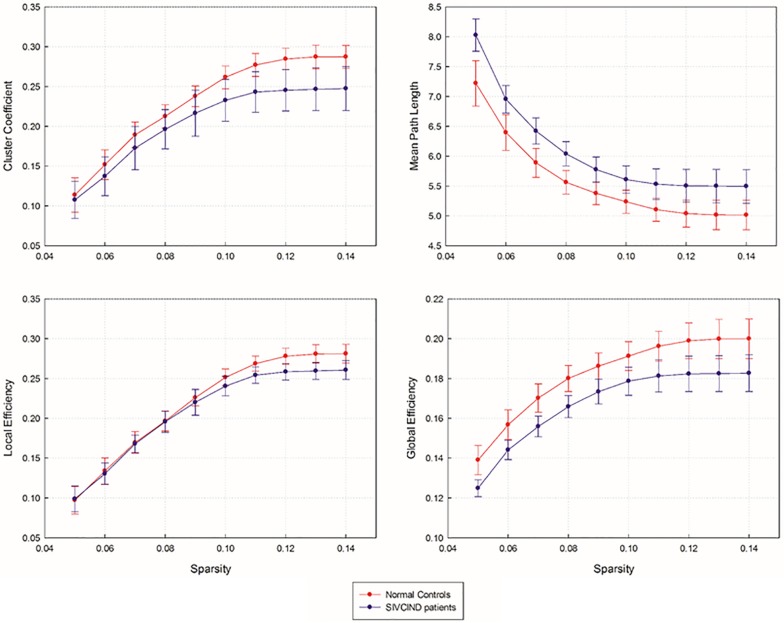
Network topological parameters of structural brain network as a function of sparsity ranging from 5 to 14%. Error bars denote standard deviations.

The SIVCIND patients showed a significantly decreased C_p_ (*p* = 0.003, FDR corrected) and global efficiency E_g_ (*p* < 0.001, FDR corrected) and a significantly increased L_p_ (*p* < 0.001, FDR corrected) compared to the normal controls as determined by the non-parametric permutation test (Figure 2). Moreover, there was no significant difference in E_bloc_ between the SIVCIND patients and the normal controls (Figure 2).

### Between-Group Differences in Nodal Efficiency

**FIGURE 3:**
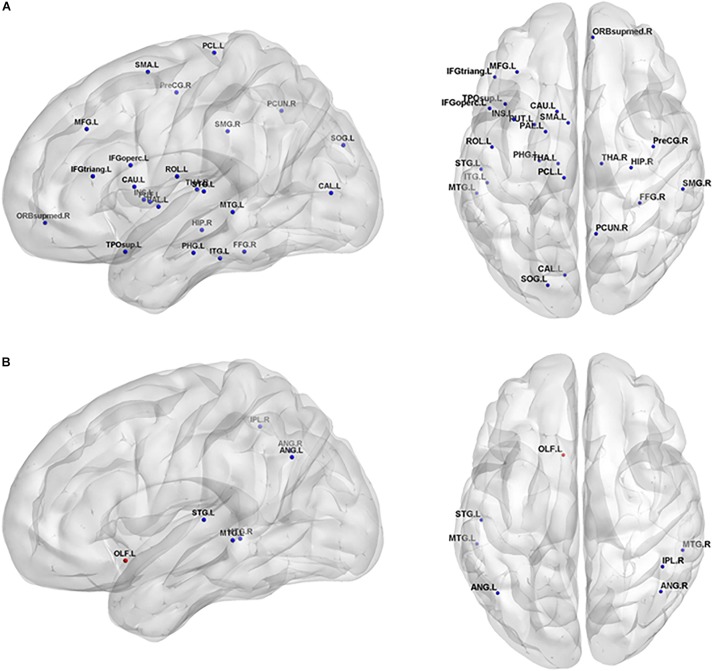
Brain regions show significant alterations of nodal efficiency in SIVCIND patients compared with normal controls (*p* < 0.05, FDR-corrected). The results were visualized using BrainNet Viewer (NKLCNL, Beijing Normal University). Three-dimensional maps show the significant differences in nodal global efficiency **(A)** and nodal local efficiency **(B)** between the SIVCIND patients and normal controls. Red/blue spheres denote regions where nodal efficiency are increased/decreased in SIVCIND patients. Detailed brain region information corresponding to the anatomical labels can be found in AAL.

The non-parametric permutation test revealed that the SIVCIND patients showed remarkably decreased E_nodal_glob_ compared with the normal controls, and only several brain regions exhibited altered nodal local efficiency (*p* < 0.05, FDR corrected) (Figure 3, and Table 2). Compared with the NCs, the SIVCIND patients showed decreased E_nodal_glob_ in prefrontal, parietal, temporal, mediotemporal and subcortical regions, such as the bilateral thalamus; the left middle frontal gyrus, inferior frontal gyrus, supplementary motor area, parahippocampal gyrus, insula, caudate nucleus, putamen, and pallidum; and the right superior frontal gyrus (orbital medial part), hippocampal gyrus, fusiform gyrus, inferior parietal gyrus, and precuneus. Moreover, the SIVCIND patients exhibited increased E_nodal_glob_ in the left olfactory cortex (Figure 3A and Table 2).

**TABLE 2:** Brain regions showing abnormal nodal efficiency in SIVCIND patients compared with normal controls (FDR-corrected *p* < 0.05 shown in bold font).

**Regions**	**Nodal global efficiency**	**Nodal local efficiency**
**SIVCIND patients < Normal controls**		
Right precentral gyrus	**0.02**	0.231
Left middle frontal gyrus	**0.017**	0.874
Left inferior frontal gyrus, operculum part	**0.001**	0.197
Left inferior frontal gyrus, triangular part	**< 0.001**	0.413
Left Rolandic operculum	**0.001**	0.316
Left supplementary motor area	**0.005**	0.183
Right superior frontal gyrus, orbital media part	**0.024**	0.363
Left insula	**0.003**	0.075
Right hippocampal gyrus	**0.045**	0.334
Left parahippocampal gyrus	**0.033**	0.789
Left calcarine cortices	**0.011**	0.553
Left superior occipital gyrus	**0.034**	0.795
Right fusiform gyrus	**0.007**	0.481
Right inferior parietal gyrus	**0.047**	**0**.**038**
Right supramarginal gyrus	**0.009**	0.591
Left angular gyrus	0.1	**0**.**018**
Right angular gyrus	0.227	**0**.**009**
Right precuneus	**0.002**	0.569
Left paracentral lobule	**< 0.001**	0.849
Left caudate nucleus	**0.034**	0.512
Left putamen	**0.012**	0.056
Left pallidum	**0.007**	0.227
Left thalamus	**0.025**	0.337
Right thalamus	**0.022**	0.654
Left superior temporal gyrus	**0.013**	**0**.**006**
Left superior temporal gyrus, pole part	**0.004**	0.271
Left middle temporal gyrus	**0.009**	**0**.**002**
Right middle temporal gyrus	0.282	**0**.**004**
Left inferior temporal gyrus	**0.02**	0.283
**SIVCIND patients > Normal controls**		
Left olfactory cortex	0.775	**0**.**007**

Compared with the NCs, the SIVCIND patients exhibited lower nodal local efficiency in the bilateral angular gyrus and middle temporal gyrus, the right inferior parietal gyrus, and the left superior temporal gyrus (Figure 3B and Table 2).

### Altered Structural Connectivity

**FIGURE 4:**
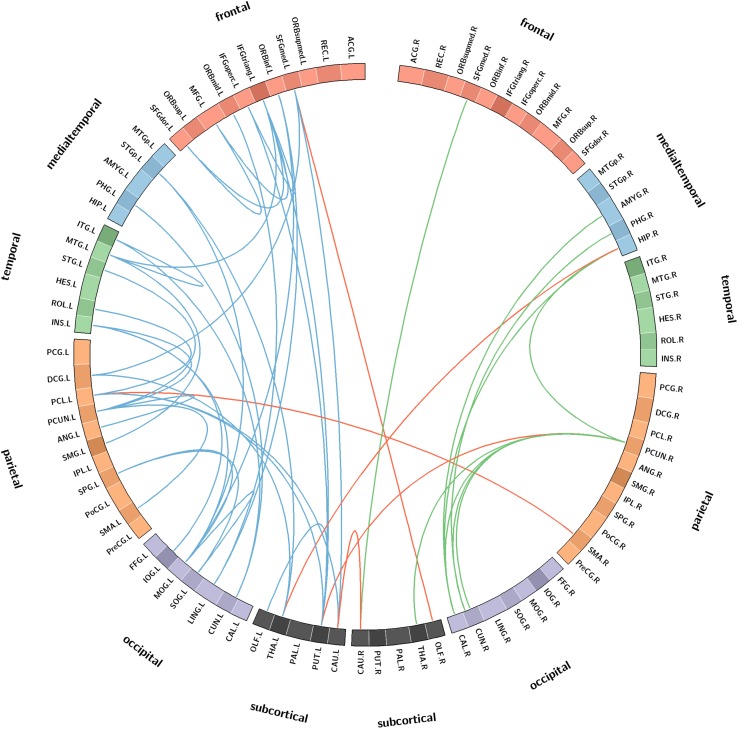
Connectograms show the decreased structural connections in SIVCIND patients compared to NCs (NBS corrected, *p* < 0.01). Links are colored by connection type as follows: left intrahemispheric (blue), interhemispheric (red) and right intrahemispheric (green). ROIs were grouped according to Salvador (Salvador et al., 2005) (i.e., frontal, temporal, parietal, medial temporal, occipital, and subcortical).

Compared with the NCs, the SIVCIND patients showed 46 significantly decreased structural connections (NBS corrected, *p* < 0.01) (Figure 4). There was an approximately 30% decrease in the structural connectivity between cortical regions and subcortical regions. Notably, most of the decreased structural connectivity in the SIVCIND patients was involved in the default mode network.

In addition, compared with the NCs, the SIVCIND patients had weaker hub connections in the bilateral precuneus; the right hippocampal gyrus and the calcarine cortices; and the left inferior frontal gyrus (triangular part), superior frontal gyrus (orbital media part), insula, lingual gyrus, middle occipital gyrus, paracentral lobule, caudate nucleus, putamen, thalamus, and middle temporal gyrus.

### Relationships Between Topological Properties and Cognitive Test Scores

**FIGURE 5:**
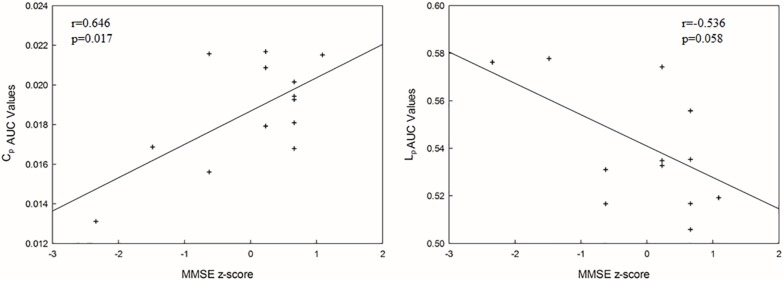
The correlations between each AUC network metric value and MMSE score (*p* < 0.05). C_p_, clustering coefficient; L_p_, mean path length.

There was a positively significant correlation between C_p_ values and the MMSE scores, and an approximately negative correlation between L_p_ values and the MMSE scores (Figure 5).

### Sensitivity and Specificity of Network Properties in Differentiating Patients From Normal Controls

Using the combination of the four network metrics (Cp, Lp, Eg, and Eloc) as predictors, a classification accuracy of 78% was obtained with a sensitivity of 69% and a specificity of 86%.

## Discussion

In this study, we investigated the altered topological properties of the structural network in SIVCIND patients. The results revealed that the C_p_ and E_glob_ decreased and the L_p_ increased in SIVCIND patients. More specifically, using the four network global parameters as features to discriminate SIVCIND patients from normal controls, a classification accuracy of 78% was obtained for all subjects, with a sensitivity of 69% and a specificity of 86%. These results implied a disturbance in information exchange in the structural brain network of SIVCIND patients. Moreover, the significantly decreased structural connectivity mainly concerned the prefrontal, temporal, parietal and subcortical cortices. Additionally, we observed several brain regions showing decreased E_nodal_glob_ in the SIVCIND patients, mostly located in the prefrontal, subcortical, and media temporal cortices. More specifically, we found significantly increased nodal local efficiency in the left olfactory cortex in the SIVCIND patients. These results provide insights into the relationship between altered structural networks and cognitive deficits in SIVCIND patients.

### Altered Global Topological Properties in SIVCIND Patients Compared With Normal Controls

As shown by the significant decrease in the values of the C_p_ and E_glob_ and significant increase in the values of the L_p_ in the SIVCIND patients, there was a disruption in the organization of the brain structural network in this cohort (Figure 2). The changes in these global network metrics could be attributable to decreased long-distance structural connections and the density of connections involved in specialized networks such as the default mode network in SIVCIND patients (Figure 4). Specifically, the MMSE scores were positively correlated with the C_p_ and had a tendency of negative correlation with L_p_ (Figure 5). Hence, the deterioration of the cognitive functioning in the SIVCIND patients was associated with less-extended local clustering and a reduced ability to transmit information from distributed brain regions. Our results were in keeping with previous studies. For instance, Du et al. (2019) found that the global network efficiency of the brain structural network was significantly related to the cognitive state in patients with mild cognitive impairment caused by small vessel disease. Moreover, Heinen et al. (2018) also found that global network efficiency of the brain structural network was related to cognitive functioning such as information processing speed, attention, and executive functioning in patients with mild cognitive impairment caused by small vessel disease.

Our results are also in accord with some previous studies regarding functional organization in SIVCIND. For example, one study found that there was decreased C_p_ and E_glob_ in several brain regions located in parietal and temporal cortices, and these changes were related to the performance on cognitive test (Yu et al., 2015). Moreover, our recent work (Sang et al., 2018) also found that SIVCIND patients showed significantly decreased E_glob_ and C_p_ in the brain functional connectivity network, and these changes were correlated with cognitive deficits.

### Decreased Nodal Efficiency in SIVCIND Compared With Normal Controls

The local (global) efficiency at the nodal level was also studied, which describes the capacity for information exchange between one node and the direct neighbors (and all other nodes in the networks). Notably, twenty-six brain regions exhibited significantly decreased E_nodal_glob_ and no brain region exhibited significantly increased E_nodal_glob_, suggesting that the capacity of information transfer was reduced between one node and the remaining nodes in the network in SIVCIND patients (Figure 3 and Table 2).

Several regions of decreased E_nodal_glob_ were found in SIVCIND patients compared with normal controls, including the prefrontal and subcortical cortex, which are the primary components of the prefrontal/subcortical circuit. Previous studies verified that the interruption of this circuit was associated with executive dysfunction, which is a specific symptom of cognitive dysfunction seen in SIVCIND patients (Román et al., 2002; Erkinjuntti, 2003). Moreover, SIVCIND has been considered to be a syndrome of extensive damage to the white matter tracts or U-fibers, which connect cortical and subcortical regions. Thus, our results are in line with previous studies.

Specifically, we found that the SIVCIND patients showed significantly increased nodal local efficiency in the left olfactory cortex (Figure 3 and Table 2). The olfactory cortex is the area responsible for receiving and processing smell-related stimuli and includes the prepyriform area and the entorhinal cortex (EC). Specifically, the EC is an important memory center in the brain. The EC forms the main input to the hippocampus and is responsible for the preprocessing of the input signals (Buzsaki and Moser, 2013), and the EC-hippocampus system plays an important role in memory consolidation and optimization in sleep. Increased nodal local efficiency of the olfactory gyrus may act as a compensation mechanism for memory deficits in SIVCIND patients.

Compared with the NCs, the SIVCIND patients had lower nodal global and local efficiency in several temporal and parietal brain areas, including the inferior parietal gyrus, superior and middle temporal gyrus. Moreover, Thong et al. (2014) found that the parietal and temporal cortices thin as cognitive impairment worsens in patients with cognitive impairment caused by SIVD, showing evidence of structural alterations in SIVCIND patients. Additionally, decreased nodal efficiency in the parietal/temporal areas suggests that their lower capacity for information exchange in the structural connectivity network is presumably associated with non-cognitive symptoms such as apathy and decreased motor function in SIVCIND patients (Gupta and Dasgupta, 2014).

### Decreased Structural Connectivity in SIVCIND

The NBS analyses found decreased structural connections in the SIVCIND patients compared with the normal controls (Figure 4). A previous structural study found cortical thinning in SIVCIND patients, and the extent and severity of the cortical thinning were in accordance with those seen in cognitive impairment (Meyer et al., 2007; Seo et al., 2010). An examination of the connectogram shows that the weakened alterations in the SIVCIND patients mainly concerned fronto-occipital, fronto-subcortical and temporo-occipital connections. Some previous DTI studies found alterations in the brain white matter between SIVCIND patients and normal controls. For instance, decreased FA value was found in several projection fibers and association fibers in SIVCIND patients, including the posterior thalamic radiations, cingulum, and fronto-occipital fasciculus (Lin et al., 2015). Moreover, Zhou et al. (2011) found decreased FA value in the bilateral frontal lobes, occipital lobes, temporal lobes, and insula in SIVCIND patients compared with normal controls. Therefore, the results from this study are in line with those previous studies, and these alterations may be responsible for cognitive impairment in SIVCIND patients. Additionally, significantly decreased connections were observed in the mediotemporal cortices in the SIVCIND patients, which is thought to be involved in encoding declarative memory (Squire et al., 2004), and decreased connections in this area can lead to forgetfulness, a clinical manifestation of cognitive impairment caused by SIVD (Román et al., 2002).

Furthermore, almost all the network hubs were components of the default mode network (DMN) (Table 3). The DMN is considered a critical resting-state functional network of the brain. Previous studies have suggested that the DMN is associated with cognitive and emotional processing (Mantini and Vanduffel, 2013). Decreased functional connections in the DMN were found in our recent study (Sang et al., 2018). Moreover, Jiang et al. (2018) also found decreased functional connections in the DMN in ischemic stroke patients. The significantly decreased structural connectivity in these brain regions may lead to functional disruption in the DMN in the functional network and may provide new insight to investigate the relationship between structural alterations and functional degeneration.

**TABLE 3:** Significant structural connectivity differences between SIVCIND patients and normal controls (*p* < 0.01, NBS corrected).

**Contrast**	**Side**	**Brain regions**
NC vs. SIVCIND		
	Right	Hippocampal gyrus Precuneus Calcarine cortices
	Left	Inferior frontal gyrus, triangular part Superior frontal gyrus, orbital media part Insula
		Lingual gyrus Precuneus Middle occipital gyrus Paracentral lobule Caudate nucleus Putamen Thalamus Middle temporal gyrus

*Only cortical and subcortical regions with a high degree of significant connections (*k* > 1 SD above the mean) are reported.*

Several limitations in our study need to be further addressed. First, although the patients enrolled in the study were been strictly checked to ensure the homogeneity of vascular lesions as far as possible, the severity degree of the lesions was not absolutely uniform across the patients. In future studies, we should recruit more patients to ensure the homogeneity of vascular lesions. Second, the sample size in this study was not large which may lead to insufficient statistical power identifying the group difference between SIVCIND patients and normal controls. Third, the study employed deterministic tractography methods to construct a brain white matter connectivity network. However, the tracking procedure would terminate when it arrived at a voxel with fiber crossings or spreading, which always reduces the tracking of existing fibers. In future studies, to obtain a more accurate assessment of white matter connectivity, we should apply more advanced diffusion acquisition techniques and diffusion models, such as probabilistic tractography (Behrens et al., 2007).

## Conclusion

Our results revealed that cognitive impairment in SIVCIND patients is associated with alterations in the structural connectivity network. The topological organization of the network in SIVCIND was disrupted, with significantly decreased C_p_ and E_glob_ and a significantly increased L_p_. Moreover, a significantly decreased E_nodal_glob_ was found in some areas that are mainly involved in the prefrontal, parietal, mediotemporal and subcortical cortices in the SIVCIND patients. Decreased structural connections were found in SIVCIND mainly concerning cortical-subcortical connectivity. These results suggest a disrupted organization in the brain structural connectivity network in SIVCIND patients; these results may be helpful to develop reliable biomarkers of structural network changes related to cognitive decline in SIVD.

## Data Availability Statement

The datasets generated for this study are available on request to the corresponding author.

## Ethics Statement

The studies involving human participants were reviewed and approved by the local Medical Ethics Committee at Third Military Medical University (Chongqing, China). The patients/participants provided their written informed consent to participate in this study.

## Author Contributions

LS, CLi, and MQ conceived and designed the experiments. LS, CLiu, LW, JZ, YZ, and PL performed the experiments. LS, JZ, and LQ analyzed the data. JZ and YZ contributed the reagents, materials, and analysis tools. LS wrote the manuscript and performed figure processing.

## Conflict of Interest

The authors declare that the research was conducted in the absence of any commercial or financial relationships that could be construed as a potential conflict of interest.

## Footnotes

1 http://www.fmrib.ox.ac.uk/fsl

2 http://trackvis.org/dtk/

3 https://www.nitrc.org/projects/gretna/

4 http://www.circos.ca
