# Supplementary material for: Disrupted Brain Structural Connectivity Network in Subcortical Ischemic Vascular Cognitive Impairment With No Dementia
Source: Front Aging Neurosci. 2020 Jan 29;12:6. doi: 10.3389/fnagi.2020.00006 (PMC7000429; doi:10.3389/fnagi.2020.00006)
Supplement: Supplementary file 1 [file Table_1.DOCX]

Disrupted Brain Structural Connectivity Network in Subcortical Ischaemic Vascular Cognitive Impairment with no dementia

Linqiong Sang^1#^, Chen Liu^2#^, Li Wang^1^, Jingna Zhang^1^, Ye Zhang^1^, Pengyue Li^1^, Liang Qiao^1^, Chuanming Li^3*^, Mingguo Qiu^1*^

^1^Department of Medical Imaging, College of Biomedical Engineering, Third Military Medical University, Chongqing, China

^2^ Department of Radiology, Southwest Hospital, Third Military Medical University, Chongqing, China

^3^ Department of Radiology, the Second Affiliated Hospital of Chongqing Medical University, Chongqing, China

^#^ These authors contributed equally to this work.

^*^Corresponding authors:

M. Qiu

E-mail address: qiumg_2002@sina.com

C. Li

E-mail address: li_chuanming@yeah.net

**Supplementary materials**

**Supplementary** **Table S1** Anatomical regions of interest (ROIs)

| Index | Regions | Abbr. | Index | Regions | Abbr. |
| --- | --- | --- | --- | --- | --- |
| 1 | Precentral_L | PreCG.L | 46 | Cuneus_R | CUN.R |
| 2 | Precentral_R | PreCG.R | 47 | Lingual_L | LING.L |
| 3 | Frontal_Sup_L | SFGdor.L | 48 | Lingual_R | LING.R |
| 4 | Frontal_Sup_R | SFGdor.R | 49 | Occipital_Sup_L | SOG.L |
| 5 | Frontal_Sup_Orb_L | ORBsup.L | 50 | Occipital_Sup_R | SOG.R |
| 6 | Frontal_Sup_Orb_R | ORBsup.R | 51 | Occipital_Mid_L | MOG.L |
| 7 | Frontal_Mid_L | MFG.L | 52 | Occipital_Mid_R | MOG.R |
| 8 | Frontal_Mid_R | MFG.R | 53 | Occipital_Inf_L | IOG.L |
| 9 | Frontal_Mid_Orb_L | ORBmid.L | 54 | Occipital_Inf_R | IOG.R |
| 10 | Frontal_Mid_Orb_R | ORBmid.R | 55 | Fusiform_L | FFG.L |
| 11 | Frontal_Inf_Oper_L | IFGoperc.L | 56 | Fusiform_R | FFG.R |
| 12 | Frontal_Inf_Oper_R | IFGoperc.R | 57 | Postcentral_L | PoCG.L |
| 13 | Frontal_Inf_Tri_L | IFGtriang.L | 58 | Postcentral_R | PoCG.R |
| 14 | Frontal_Inf_Tri_R | IFGtriang.R | 59 | Parietal_Sup_L | SPG.L |
| 15 | Frontal_Inf_Orb_L | ORBinf.L | 60 | Parietal_Sup_R | SPG.R |
| 16 | Frontal_Inf_Orb_R | ORBinf.R | 61 | Parietal_Inf_L | IPL.L |
| 17 | Rolandic_Oper_L | ROL.L | 62 | Parietal_Inf_R | IPL.R |
| 18 | Rolandic_Oper_R | ROL.R | 63 | SupraMarginal_L | SMG.L |
| 19 | Supp_Motor_Area_L | SMA.L | 64 | SupraMarginal_R | SMG.R |
| 20 | Supp_Motor_Area_R | SMA.R | 65 | Angular_L | ANG.L |
| 21 | Olfactory_L | OLF.L | 66 | Angular_R | ANG.R |
| 22 | Olfactory_R | OLF.R | 67 | Precuneus_L | PCUN.L |
| 23 | Frontal_Sup_Medial_L | SFGmed.L | 68 | Precuneus_R | PCUN.R |
| 24 | Frontal_Sup_Medial_R | SFGmed.R | 69 | Paracentral_Lobule_L | PCL.L |
| 25 | Frontal_Mid_Orb_L | ORBsupmed.L | 70 | Paracentral_Lobule_R | PCL.R |
| 26 | Frontal_Mid_Orb_R | ORBsupmed.R | 71 | Caudate_L | CAU.L |
| 27 | Rectus_L | REC.L | 72 | Caudate_R | CAU.R |
| 28 | Rectus_R | REC.R | 73 | Putamen_L | PUT.L |
| 29 | Insula_L | INS.L | 74 | Putamen_R | PUT.R |
| 30 | Insula_R | INS.R | 75 | Pallidum_L | PAL.L |
| 31 | Cingulum_Ant_L | ACG.L | 76 | Pallidum_R | PAL.R |
| 32 | Cingulum_Ant_R | ACG.R | 77 | Thalamus_L | THA.L |
| 33 | Cingulum_Mid_L | DCG.L | 78 | Thalamus_R | THA.R |
| 34 | Cingulum_Mid_R | DCG.R | 79 | Heschl_L | HES.L |
| 35 | Cingulum_Post_L | PCG.L | 80 | Heschl_R | HES.R |
| 36 | Cingulum_Post_R | PCG.R | 81 | Temporal_Sup_L | STG.L |
| 37 | Hippocampus_L | HIP.L | 82 | Temporal_Sup_R | STG.R |
| 38 | Hippocampus_R | HIP.R | 83 | Temporal_Pole_Sup_L | TPOsup.L |
| 39 | ParaHippocampal_L | PHG.L | 84 | Temporal_Pole_Sup_R | TPOsup.R |
| 40 | ParaHippocampal_R | PHG.R | 85 | Temporal_Mid_L | MTG.L |
| 41 | Amygdala_L | AMYG.L | 86 | Temporal_Mid_R | MTG.R |
| 42 | Amygdala_R | AMYG.R | 87 | Temporal_Pole_Mid_L | TPOmid.L |
| 43 | Calcarine_L | CAL.L | 88 | Temporal_Pole_Mid_R | TPOmid.R |
| 44 | Calcarine_R | CAL.R | 89 | Temporal_Inf_L | ITG.L |
| 45 | Cuneus_L | CUN.L | 90 | Temporal_Inf_R | ITG.R |

The regions are listed in terms of a prior AAL atlas. Abbr., abbreviations.


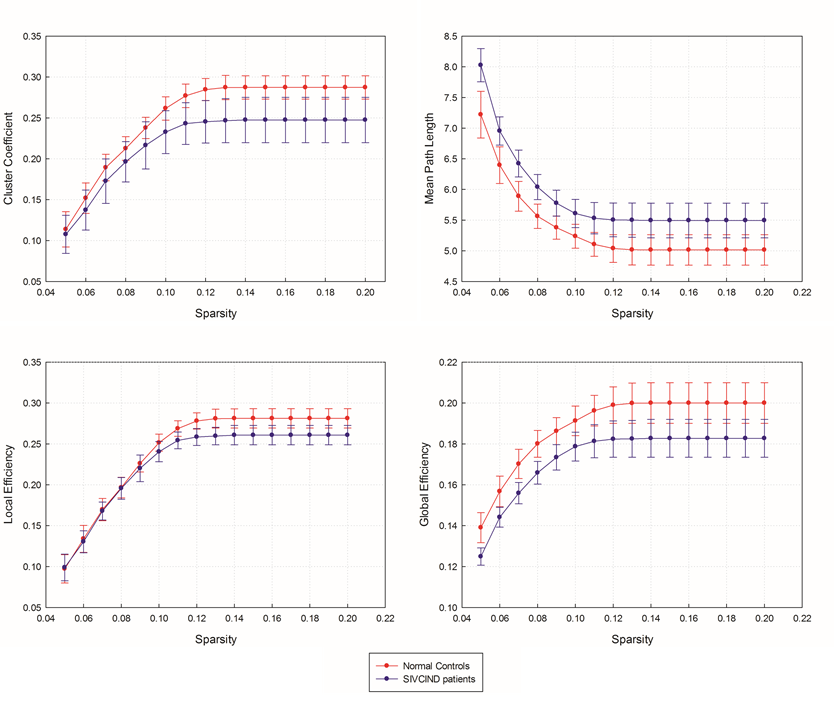


**Supplementary** **Figure S1.** Network topological parameters of functional brain networks as a function of sparsity for SIVCIND patients and normal controls ranging from 5% to 20%. Error bars denote standard deviations.
